# Inferring Gene Regulatory Networks From Single-Cell Transcriptomic Data Using Bidirectional RNN

**DOI:** 10.3389/fonc.2022.899825

**Published:** 2022-05-26

**Authors:** Yanglan Gan, Xin Hu, Guobing Zou, Cairong Yan, Guangwei Xu

**Affiliations:** ^1^School of Computer Science and Technology, Donghua University, Shanghai, China; ^2^School of Computer Engineering and Science, Shanghai University, Shanghai, China

**Keywords:** gene regulatory network, recurrent neural network, gene expression, single-cell transcriptomic data, bidirectional structure

## Abstract

Accurate inference of gene regulatory rules is critical to understanding cellular processes. Existing computational methods usually decompose the inference of gene regulatory networks (GRNs) into multiple subproblems, rather than detecting potential causal relationships simultaneously, which limits the application to data with a small number of genes. Here, we propose BiRGRN, a novel computational algorithm for inferring GRNs from time-series single-cell RNA-seq (scRNA-seq) data. BiRGRN utilizes a bidirectional recurrent neural network to infer GRNs. The recurrent neural network is a complex deep neural network that can capture complex, non-linear, and dynamic relationships among variables. It maps neurons to genes, and maps the connections between neural network layers to the regulatory relationship between genes, providing an intuitive solution to model GRNs with biological closeness and mathematical flexibility. Based on the deep network, we transform the inference of GRNs into a regression problem, using the gene expression data at previous time points to predict the gene expression data at the later time point. Furthermore, we adopt two strategies to improve the accuracy and stability of the algorithm. Specifically, we utilize a bidirectional structure to integrate the forward and reverse inference results and exploit an incomplete set of prior knowledge to filter out some candidate inferences of low confidence. BiRGRN is applied to four simulated datasets and three real scRNA-seq datasets to verify the proposed method. We perform comprehensive comparisons between our proposed method with other state-of-the-art techniques. These experimental results indicate that BiRGRN is capable of inferring GRN simultaneously from time-series scRNA-seq data. Our method BiRGRN is implemented in Python using the TensorFlow machine-learning library, and it is freely available at https://gitee.com/DHUDBLab/bi-rgrn.

## 1 Introduction

Gene regulatory mechanisms are crucial to understanding diverse dynamic processes such as development, stress response and disease (1). Cell states and the dynamics of cell behavior are governed by complex gene interactions (2), which in turn define cellular morphology and functions. Such regulatory interactions can be modeled as a gene regulatory network (GRN), where nodes are regulators and their target genes, and edges represent the regulatory relationships between genes (3). Unraveling GRNs is one of the major challenges in the field of computational biology, which allows us to pinpoint key factors that determine phenotype in health systems as well as in diseases (4, 5).

A plethora of computational or statistical approaches have been developed for inferring networks from observational gene expression data (6–8). The widely used algorithm GENIE3 decomposes the inference of gene regulatory networks into different regression subproblems. Using tree-based ensemble methods, the expression pattern of each target gene is predicted by the expression of all the other genes (9). ENNET also considers the inference problem as a regression task, which is solved by a decision tree optimizing the least-squares loss function (10). It builds the model additively using a boosting procedure. PPCOR reconstructs gene regulatory network by calculating partial correlation coefficient and semi-partial correlation coefficient between genes (11). PIDC exploits information theory to infer the regulatory relationship between genes (12). Biologically, it is assumed that changes in regulators should precede changes in their targets in time. However, such time information is not available in steady-state gene expression data, and thus GRNs constructed from these data have limited ability to capture dynamic regulatory relationships between genes. Several methods have been proposed to infer GRNs based on time-series gene expression data to address this issue. The algorithm LEAP reconstructs gene regulatory networks by calculating the Pearson correlation coefficient. With pseudo-time data information, the algorithm defines a fixed-size time window and assumes that the earlier expressed gene in this window can affect other genes (13). SCODE infers regulatory networks based on ordinary differential equations and linear regression (14). The method SINCERITIES adopts the Kolmogorov–Smirnov distance to quantify the distance between two cumulative distribution functions of gene expressions from subsequent time points, and recovers directed regulatory relationships among genes by employing regularized linear regression (15). BiXGBoost infers the regulatory network through both forward and reverse directions, separately considering the regulatory genes and target genes of specific genes, and uses the gradient boosting decision tree to integrate the final regulatory relationship (16). The algorithm GRGNN proposes an end-to-end gene regulation graph neural network approach to reconstruct GRNs from scratch utilizing gene expression data in both a supervised and a semi-supervised framework (17). DeepSEM is a neural network version of the structural equation model (SEM) to explicitly model the regulatory relationships among genes (18). These efforts mainly focus on intracellular interactions, inferring gene regulatory relationships within a specific cell. Recently developed methods for spatial transcriptomics are now providing high-throughput information about both the expression patterns of genes within a single cell and the spatial relationships between cells (19–21). The algorithm CNNC is a supervised framework for gene relationship inference, using convolutional neural networks to analyze summarized co-occurrence histograms from pairs of genes in scRNA-seq data (22). GCNG transforms the problem of gene regulation network reconstruction into a classification problem. It uses a graph convolutional neural network to fit cell location information and gene expression data and infer the final result (23).

Although much progress has been made, inferring a network of regulatory interactions between genes is still challenging. On one hand, for time-series scRNA-seq data, methods for reconstructing GRNs on bulk data are not directly applicable. As the biological meaning of a sample changes from the average for several cells in bulk data to the value for a single cell, the form of the gene expression data is also changed. Meanwhile, as the approaches devised for single-cell transcriptomics typically require a large number of time points to infer GRNs, they are usually suitable for a small number of genes. Adding a few genes to a network inference analysis may require the inference algorithm to consider many additional regulatory interactions between them. As the number of genes grows, the number of edges and the demand for input data might explode.

Here, we present BiRGRN, a novel method of inferring GRNs from time-series scRNA-seq data. BiRGRN adopts a bidirectional recurrent neural network to infer GRNs. The recurrent neural network is a deep neural network that can capture complex, non-linear, and dynamic relationships among variables. It maps a neuron to a gene, and maps the connections between neural network layers to the regulatory relationship between genes, giving a good solution to model GRN with biological closeness and mathematical flexibility. Then we transform the reconstruction of GRNs into a regression problem, using the gene expression data of the previous time points to predict the gene expression data of the later time point. Meanwhile, we adopt a bidirectional structure and incorporate an incomplete set of prior knowledge to improve the accuracy and stability of the algorithm. To evaluate the performance of BiRGRN, we apply it to four simulated datasets and three real single-cell transcriptomic datasets. We performed a comparison of our results with other state-of-the-art techniques, which shows the better performance of our proposed model.

## 2 Materials

### 2.1 The BiRGRN Method

**Figure 1:**
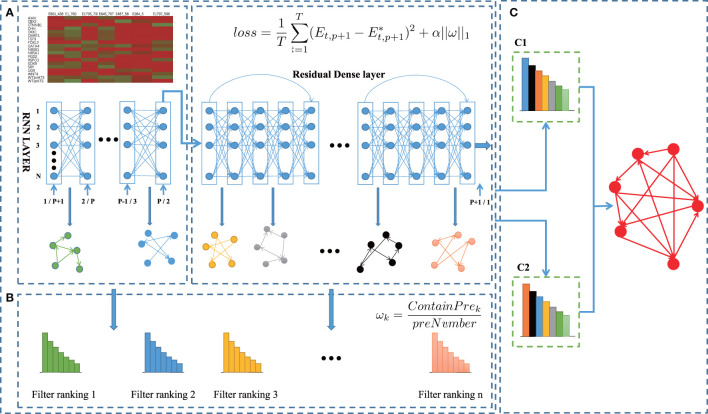
BiRGRN reconstructs GRNs from time-series single cell transcriptome data using bidirection RNN. **(A)** Inferring initial gene regulatory network with RNN. **(B)** Incorporating incomplete prior knowledge to adjust candidate regulatory edges. **(C)** Adopting a voting strategy to integrate multiple candidate regulatory networks, and further utilizing bidirectional model to optimize the inferred GRN.

In this work, we propose a new computational method BiRGRN to reconstruct gene regulatory networks based on bidirectional recurrent neural network and multiple prior networks. The overview of the BiRGRN is shown in **Figure 1**. The proposed algorithm consists of the following three main steps. Firstly, we train a deep neural network to infer preliminary gene regulatory networks, where neurons are mapped to genes, and the links between adjacent layers of the neural network are related to gene regulation relationships. Secondly, we incorporate incomplete prior knowledge to filter the candidate regulatory edges obtained in the first step. Finally, we adopt a voting strategy to integrate multiple candidate regulatory networks and utilize a bidirectional strategy to optimize the inferred GRN.

#### 2.1.1 Step 1: Training RNN to Infer the Initial Gene Regulatory Networks

Inferring gene regulatory network from single-cell transcriptomic data is actually to construct a directed graph, where the nodes represent the genes, and the edges represent the regulatory relationships among genes. If we assume that the expression pattern of gene *i* at time point *p+1* is the total regulatory effect of the expression values of all genes at the previous *p* time points, the regulation process can be described as the following function (16):


(1) $$e_{p+1}^{i}=f^{i}\left(E_{p}\right)+\in_{i}$$

where $e_{p+1}^{i}$ represents the expression value of gene *i* at the time point *p+1*, *E_p_* represents the expression value of all genes at the previous *p* time points, and ∈*_i_* represents the influence of external noise. Specifically, *p* is the time lag, which represents the maximum time delay of the interaction between genes.

**Figure 2:**
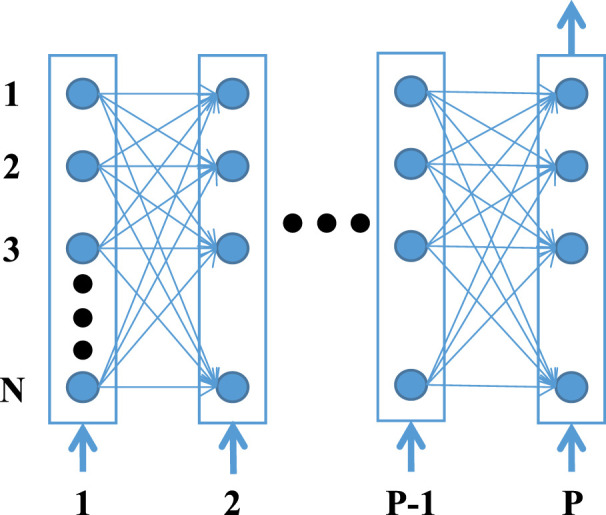
The schematic structure of a RNN unfolded in time. Each node corresponds to a gene and a connection between two nodes defines their interaction.

Here, to model the regulation process of different genes in a parallel manner, we adopt RNN to formalize gene regulatory networks (24). A recurrent neural network is a type of artificial neural network that can capture complex, non-linear, and dynamic relationships among variables. It is mainly used for processing sequential data like time series and solving ordinal or temporal problems. As shown in the example RNN (**Figure 2**), each node represents a particular gene and the edges between the nodes represent the regulatory interactions among the genes. Each layer of the neural network defines the gene expression level of the genes at a specific time point. The expression level of all genes at the time point *p+1* depends upon the expression level of all the genes at the preceding *p* time points and the weights of the corresponding connecting edges with that particular gene (25, 26). Then the regulation process can be formulated as:


(2) $$E_{p+1}=F\left(E_{p}\right)+\in$$

where *E_p_*_+1_ represents the expression value of all genes at the time point *p+1*.

To improve the stability of the algorithm, BiRGRN integrates multiple fully connected layers with the RNN to train gene expression data. Therefore, the proposed network structure consists of an RNN, multiple fully connected layers with ResNet residual connections (27), and an output layer. In detail, the proposed RNN contains *p* layers corresponding to *p* time points, with multiple inputs and one output. Subsequently, the output of the RNN is used as the input of these fully connected layers. To avoid the over-fitting problem usually caused by the deep neural network, BiRGRN adds a ResNet residual connection for every five fully connected layers. In the experiment, we set the number of the connected layers ranging from 10 to 100. We find that too few fully connected layers will lead to a significant decrease in the stability of the algorithm, whereas too many fully connected layers can not improve the accuracy but increase the running time of the algorithm. Therefore, we use 50 fully connected layers and add a ResNet structure. To train the deep neural network, we take the gene expression data of the genes at the previous *p* time points as input, and the gene expression data at the *p+1* time point as output. Then, the problem is transformed into a supervised regression problem, which overcomes the difficulty of obtaining training labels.

Here, we utilize mean square loss (MSE) as the regression loss function for deep neural network training. The RNN is a fully connected structure, whereas the regulatory network is usually sparsely connected. Thus, we add L1 regularization in the objective function, aiming to control the sparsity of the resulted weight matrix *w*. The loss function is defined as follows:


(3) $$loss=\frac{1}{T}\sum_{t=1}^{T}{\left(E_{t,p+1}-E_{t,p+1}^{*}\right)}^{2}+\alpha {∥\omega ∥}_{1}$$

where $E_{t,p+1}^{*}$ and *E_t_*,*_p_*_+1_ respectively represent the predicted and the real expression value of all genes at the time point *t+p+1*. *α*∥ *ω* ∥_1_ is the regularized term.

For the training process, when the objective function converges to the minimum, the algorithm extracts the multiple weight matrixes between the RNN layer and each fully connected layer. Then we normalize each basic weight matrix separately. According to the proposed network structure, the weight matrix corresponds to the regulatory relationships among genes, which can be used to reconstruct a candidate gene regulatory network. For each matrix, we take the top *m* (Usually 1.2 times the number of inferred regulation edge) connections as the candidate regulatory edges. As multiple weight matrixes are obtained after the training process, we can infer multiple candidate gene regulatory networks, which are used as the basic voters to determine the final regulatory edges in the following steps.

#### 2.1.2 Step 2: Incorporating Prior Knowledge to Adjust Candidate Regulatory Edges

During the above training process, the final loss function of the model usually cannot be completely reduced to zero due to the influence of external noise. Meanwhile, in convex optimization problems, there are a large number of approximate solutions near the global optimal. In order to improve the accuracy of the GRN inference, some prior knowledge can be utilized to filter the candidate regulatory edges. The previous method, such as NetREX and MiPGRN, assumes that the prior network and the target GRN have some similarity, and then bias the optimization procedure toward networks that overlap with the prior (28, 29). Here, if the initial candidate GRN defined by the basic weight matrix has more overlap with the prior network, it is considered to be closer to the final inferred GRN. Correspondingly, this candidate GRN is assigned a higher voting weight in the following ensemble process. Specifically, the weight of the candidate GRN is calculated according to the following strategy:


(4) $$\omega_{k}=\frac{ContainPre_{k}}{preNumber}$$

where *ω_k_* represents the weight of the *k_th_* initial GRN, *ContainPre_k_* denotes the number of candidate edges in the *k_th_* inferred GRN overlapping with the prior network, and *preNumber* represents the number of the prior edges.

As the usable prior knowledge usually does not exist for given datasets, here we adopt a general strategy to obtain an incomplete prior edge set. We utilize different computational algorithms to predict the putative GRNs, apply the method NETRex to optimize the predictions, and then integrate the top 10% of the resulted edges to obtain an incomplete prior edge set (29). Through evaluating different methods, here we select three methods, including GRNBOOST2, PPCOR, and PIDC. These three methods respectively adopt a different strategy to predict GRNs. NetRex is an algorithm based on Network Component Analysis (NCA) to optimize the predicted GRN (28).

#### 2.1.3 Step 3: Utilizing a Bidirectional Model to Optimize the Inferred GRN

Based on the deep neural network, we obtain *K* candidate GRNs, and each candidate GRN possesses an adjusted weight matrix. Next, we integrate these *K* different initial gene regulatory networks. The voting strategy is the addition of weights, and finally a global regulatory edge ranking is obtained according to the weights. For the regulatory edge of gene *i* to gene *j*, the weight *e_ij_* is calculated as:


(5) $$e_{ij}=\sum_{k=1}^{K}\omega_{k}*e_{ij}^{k}$$

where *ω_k_* represents the weight of the *k_th_* candidate GRN, and $e_{ij}^{k}$ represents the regulatory edge of gene *i* to gene *j* in the *k_th_* candidate GRN.

Inspired by the bidirectional model of the algorithm BiXGBoost (16), we further utilize the bidirectional model to fully mine the regulatory genes and target genes. Different from BiXGBoost which proposes local_in and local_out models to deal with forward and reverse inference, we use forward time-series expression data and reverse time-series expression data to respectively infer two regulatory networks. For the reverse time series data, the weight matrix obtained by the model represents the regulatory strength between gene. Next, considering the directionality of the regulatory relationship, we assume that genes expressed at earlier time points regulate genes expressed at later time points. Therefore, for the reverse inference, the input of the algorithm is the gene expression data at *p* time points of *p+1*, *p*, *p-1*,…, *2*, and the output is the gene expression data of the first time point. After getting the trained model, the algorithm extracts the weight matrix *ω^r^*, and the subsequent operations are consistent with the forward model. Then the algorithm will eventually get two regulatory networks, and also use voting strategies to integrate forward and reverse results to get the final inferred regulatory network:


(6) $$e_{ij}^{*}=e_{ij}^{f}+e_{ij}^{r}$$

where $e_{ij}^{f}$ represents the weight *e_ij_* obtained from forward inferring, and $e_{ij}^{r}$ represents the weight *e_ij_* obtain the reverse inferred GRN. Based on the calculated new weights of these edges, we rank the regulatory edges and select the top *m* regulatory edges to form the inferred GRN.

### 2.2 Datasets

Real scRNA-seq data sets. In order to evaluate the performance of the proposed algorithm on real scRNA-seq datasets, we select three widely used scRNA-seq data sets as the previous method SCODE did (14). The first dataset is derived from primitive endoderm (PrE) cells differentiated from mouse ES cells (measured at 0, 12, 24, 48, and 72 hours, respectively) and contains 456 cells (30). The second dataset is derived from examining direct reprogramming from mouse embryonic fibroblast (MEF) cells to myocytes (measured on 0, 2, 5, and 22 days), and this data set contains 405 cells (31). The third dataset is the scRNA-seq data of definitive endoderm cells derived from human ES cell differentiation (measured at 0, 12, 24, 36, 72, and 96 hours, respectively), and this dataset contains 758 cells (32). In order to verify the inferred GRN on these scRNA-seq datasets, SCODE used the transcription factor regulation network database (http://www.regulatorynetworks.org), which was constructed from DNaseI footprints and TF-binding motifs (33, 34). They integrated the TF regulatory networks of human and mouse, and extracted 100*100 TF regulatory networks for each dataset. We use this regulatory network as the correct network for each data set, and calculate the AUC value of the inferred network.

Simulated data sets. For real single-cell gene expression datasets, it is usually difficult to obtain the real labels for the edges in the gene regulatory network. In order to verify the effectiveness of the proposed method and compare it with existing methods, four simulated datasets are also used to evaluate the inferred results (6). These four data sets are all generated by the Boolean model simulating real cell expression data (35). The advantage of using the Boolean model is that it can be used as a real biological regulatory network to evaluate the performance of the reconstructed regulatory network. We utilize the four gene expression data sets of gonadal sex determination (GSD), hematopoietic stem cell differentiation (HSC), ventral spinal cord development (VSC), and mammalian cortical development (mCAD) to evaluate the performance of the algorithm. These four datasets all contain 10 simulation subsets composed of 2000 cells. The detailed information of the data sets is shown in **Table 1**.

**Table 1:** Details of time-seris gene expression datasets used in the experiment.

Dataset	Genes	Time points	Cells
GSD	19	734	2000
HSC	11	731	2000
VSC	8	492	2000
mCAD	5	492	2000
Real Dataset1	100	456	456
Real Dataset2	100	405	405
Real Dataset3	100	758	758

### 2.3 Evaluation Metrics

To evaluate the performance of different methods in inferring GRNs, we utilize two widely-used metrics AUROC and AUPRC. Specifically, AUROC is the area under the ROC based on TPR and FPR. AUPRC is the area under the PRC based on the precision rate and the recall rate.


(7) $$TPR=\frac{TP}{TP+FN}$$

(8) $$FPR=\frac{FP}{FP+TN}$$

(9) $$Precision=\frac{TP}{TP+FP}$$

(10) Recall=TPR

where TP and FP indicate the numbers of true and false positives, and TN and FN are true and false negatives. For the simulated datasets, we calculated the average of the AUROC and AUPRC to evaluate the accuracy of the inferred network on different subsets. Further, we calculated the overall score of *AUROC_score_* and *AUPRC_score_*. The definition is as follows:


(11) $$AUROC_{score}=\frac{1}{n}\sum_{i=1}^{n}AUROC_{i}$$

(12) $$AUPRC_{score}=\frac{1}{n}\sum_{i=1}^{n}AUPRC_{i}$$

where *n* represents the number of subsets in each dataset (taking the dataset GSD as an example, *n* is 10). *AUROC_i_* and *AUPRC_i_* respectively denote the average AUROC and AUPRC of the algorithm on the *i_th_* data set.

## 3 Results

### 3.1 Performance on Simulated Data Sets

To evaluate the effectiveness of BiRGRN, We apply the proposed GRN inference method to four simulated datasets, including datasets related to hematopoietic stem cell differentiation (HSC), gonadal sex determination (GSD), ventral spinal cord development (VSC), and mammalian cortical development (mCAD). In detail, each dataset is generated by the Boolean model in previous study (6), including 10 data subsets composed of 2000 cells and multiple time points. **Table 1** lists the detailed information of these datasets. We take each synthetic network as the ground truth and adopt two metrics to evaluate the inferred GRNs. We utilize both the area under the receiver operating characteristic curve and the area under the precision-recall curve (AUROC/AUPRC) as our evaluation metrics across the 10 different datasets. Further, we compare BiRGRN with four widely used methods, including three prior algorithms GRNBOOST2 (36), PPCOR, PIDC, and the classic algorithm GEINE3.

**Figure 3:**
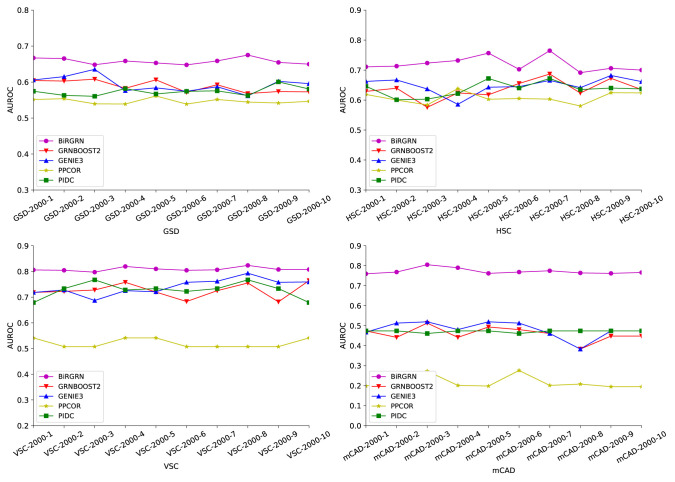
AUROC scores of the compared GRN inference algorithms on four simulated datasets.

**Figure 4:**
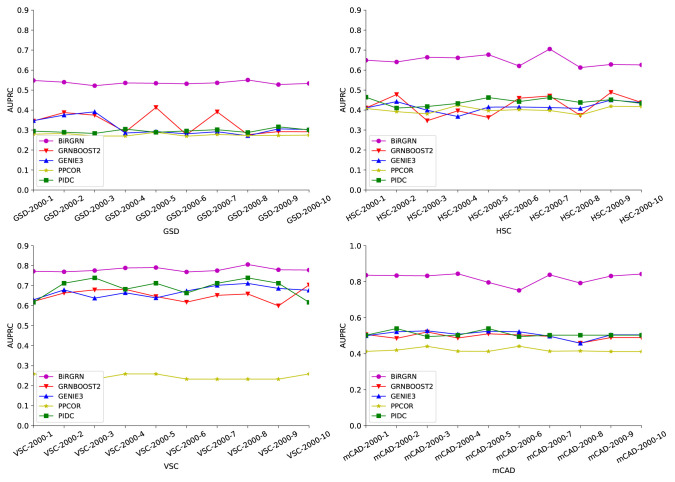
AUPRC scores of of the compared GRN inference algorithms on four simulated datasets.

**Figure 5:**
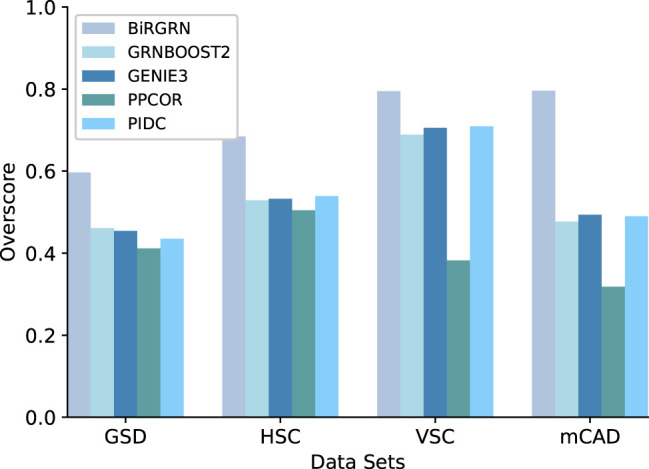
The overall score of the algorithm on the four simulated datasets.

**Figures 3**, **4** respectively show the AUROC and AUPRC of these compared methods on the four datasets. As can be seen, BiRGRN outperforms the compared methods on all four simulated datasets. We observe significant improvement over the three methods (GRNBOOST2, PPCOR, and PIDC) using the provided prior edge sets. Also, BiRGRN performs better than the widely used method GENIE3. Compared with the second-ranked algorithm on GSD, BiRGRN has a 6.2% increase in AUROC and a 33.3% increase in AUPRC. On the dataset HSC, BiRGRN achieves an improvement of 11.3% in AUROC and 10.2% in AUPRC over the other methods. For the dataset VSC, BiRGRN has a 3.8% higher AUROC and a 13.2% higher AUPRC than the second-ranked algorithm, whereas the performance of PPCOR is not as good as other methods. And as shown in the figures, the compared algorithms perform poorly on mCAD, and the AUROC values of the four algorithms are only around 0.5. In contrast, our proposed BiRGRN reaches a mean AUROC of 0.8. Compared with the second-ranked algorithm, the AUROC of BiRGRN increases by 55%, AUPRC increases by 56.1%. Furthermore, **Figure 5** presents the overall score of these algorithms on the four datasets, the histogram of the overall score also intuitively shows that the algorithm in this paper has a better performance.

### 3.2 Performance on the Real scRNA-Seq Data Sets

We next measure the performance of BiRGRN for inferring GRNs on real datasets. Here, BiRGRN is applied to three real time-series scRNA-seq datasets. As previous studies did (14), the inferred GRN is validated by the TF regulatory network based on DNaseI footprints and TF-binding motifs. We calculate the AUROC values of BiRGRN given 15% of the prior knowledge and compared them with four widely used methods, including GENIE3, LEAP, BiXGBoost, and SCODE. Specifically, GENIE3 is a classic random forest-based method for inferring GRNs. The algorithm BiXGBoost adopts local-in and local-out models to utilize time information in two directions and integrates XGBoost to evaluate the feature importance. LEAP and SCODE are two advanced GRN inference methods for scRNA-seq data.

**Table 2** presents the performance of these compared methods on the real scRNA-seq datasets. Compared to other network inference algorithms, our proposed algorithm BiRGRN can infer TF regulatory networks with high performance. On Dataset 1 and Dataset 3, the AUROC values of BiRGRN are obviously higher than those of the four previous algorithms. Compared with the second-ranked algorithm SCODE, the AUROC of BiRGRN is increased by 6.5% on dataset1, and the AUROC of BiRGRN is increased by 7.4% on dateset3. On Dataset 2, the performance of BiRGRN is close to the best performance. These results indicate that the RNN structure utilized in BiRGRN has a high capability of incorporating time point information, which is effective in network inference.

**Table 2:** The AUROC value of the algorithm on three real scRNA-seq datasets.

Algorithm	Dataset 1	Dataset 2	Dataset 3
BiRGRN	**0.571**	0.573	**0.562**
GENIE3	0.503	0.498	0.507
LEAP	0.487	0.5	0.494
SCODE	0.536	**0.581**	0.523
BiXGBoost	0.509	0.479	0.510

The value in bold represents the highest value in the column.

We also record the runtime of each method on three real data sets. As shown in **Table 3**, LEAP and GENIE3 have the highest efficiency. The runtime of BiRGRN is at the median level among several methods. On Dataset 1 and Dataset 2, BiRGRN runs for 1min and 58s, which is much faster than SCODE and BiXGBoost. These results show that BiRGRN can efficiently use temporal information to rapidly reconstruct gene regulatory networks.

**Table 3:** The runtime of each method for three real datasets.

Runtime^1^	BiRGRN	SCODE	GENIE3	LEAP	BiXGBoost
Dataset 1	1min58s	7min3s	58s	6s	min49s
Dataset 2	1min58s	6min39s	52s	4s	3min21s
Dataset 3	2min22s	8min49s	1min6s	11s	3min58s

^1^All algorithms except BiXGBoost are tested on Beeline(a benchmarking software for GRN inference algorithms). The computations were performed on a Lenovo Legion R7000 2020 equipped with a 3.0GHz AMD Ryzen 5 4600H processor a 4GB NVIDIA GeForce GTX 1650Ti and 16GB of 3200MHz DDR4 RAM.

### 3.3 Ablation Study

As BiRGRN is mainly composed of the bidirectional RNN integrating the forward and reverse training, and the voting model incorporating prior knowledge, we further investigate the impact of the different components on the overall performance. Accordingly, we obtain three variants of BiRGRN, including BiRGRN-Prior(the model removing incorporated prior knowledge), BiRGRN-Forward (the model removing forward training), and BiRGRN-Reverse (the model removing reverse training). We respectively carry out the ablation study on the four simulated datasets. **Table 4** summarizes the performance comparison between BiRGRN and these three variants.

**Table 4:** The AUROC value of the algorithm and three variants on the simulated datasets.

Dataset	BiRGRN	Prior network	Forward	Reverse
GSD	**0.597**	0.544	0.583	0.587
HSC	**0.684**	0.586	0.656	0.660
VSC	**0.795**	0.624	0.761	0.763
mCAD	0.796	0.678	0.796	0.792

The value in bold represents the highest value in the row.

We first evaluate the contribution of prior information for guiding the voting process in the model. The results show that the removal of the prior information results in a slight drop in performance. Without incorporating prior information, the network is able to reconstruct a relatively coarse segmentation. Without further guidance of prior information, it might be not able to refine it properly. To further inspect the effectiveness of the bidirectional model, we respectively compare the performance of the BiRGRN without forwarding training and reverse training. From the table, we observe that the performance of two single directional training models is similar, and they are slightly lower than those of the bidirectional training model. This result of ablation Study indicates the forward training and the reverse training might be complementary to each other, and thus the bidirectional RNN structure is capable of capturing more regulation relationships among genes. On the whole, these results demonstrate that both the components are contributive to the performance of BiRGRN.

## 4 Conclusion

Many cellular processes, either in development or disease progression are governed by complex gene regulatory mechanisms. GRN reverse engineering methods attempt to infer GRNs from large-scale transcriptomic data using computational or statistical models. A plethora of GRN inference methods has been proposed. However, with the development of single-cell sequencing technology, traditional GRN inference methods designed for bulk transcriptomic data might be unsuitable to process large quantities of scRNA-seq data. In this paper, we proposed a novel computational method BiRGRN to infer GRNs from time-series scRNA-seq data. BiRGRN utilizes a bidirectional recurrent neural network to infer GRNs. The recurrent neural network is a complex neural network, which can capture complex, non-linear, and dynamic relationships among variables. It maps a neuron to a gene, and maps the connections between neural network layers to the regulatory relationship between genes, giving a good solution to model GRN with biological closeness and mathematical flexibility. Then we transform the reconstruction of GRNs problem into a regression problem that uses the gene expression data of the previous time points to predict the gene expression data of the later time node. In order to improve the accuracy of the algorithm, the method can use an incomplete set of prior knowledge. The developed model has been tested on four simulated data and three real datasets. We performed a comparison of our results with other state-of-the-art techniques which shows the superiority of our proposed model. The experiments conducted on simulated datasets and real scRNA-seq datasets demonstrate that BiRGRN can infer gene regulatory networks with high performance, which that the proposed bidirectional RNN structure is effective in GRN inference.

## Data Availability Statement

Publicly available datasets were analyzed in this study. The real dataset can be found in https://github.com/hmatsu1226/SCODE, the simulated datasets are all from Beeline and can be found in https://github.com/Murali-group/Beeline.

## Author Contributions

YG and XH are responsible for the main idea, as well as the completion of the manuscript. XH has developed the algorithm and performed data analysis. GZ, CY, and GX have coordinated data preprocessing and supervised the effort. All authors have read and approved the final manuscript.

## Funding

This work was sponsored in part by the National Natural Science Foundation of China (62172088), National Key Research and Development Program of China (2016YFC0901704), and Shanghai Natural Science Foundation (21ZR1400400, 19ZR1402000).

## Conflict of Interest

The authors declare that the research was conducted in the absence of any commercial or financial relationships that could be construed as a potential conflict of interest.

## Publisher’s Note

All claims expressed in this article are solely those of the authors and do not necessarily represent those of their affiliated organizations, or those of the publisher, the editors and the reviewers. Any product that may be evaluated in this article, or claim that may be made by its manufacturer, is not guaranteed or endorsed by the publisher.
